# Inequity in psychiatric healthcare use in Australia

**DOI:** 10.1007/s00127-022-02310-1

**Published:** 2022-07-09

**Authors:** Rubayyat Hashmi, Khorshed Alam, Jeff Gow, Khurshid Alam, Sonja March

**Affiliations:** 1 Centre for Health Research, and School of Business, Faculty of Business, Education, Law and Arts, University of Southern Queensland, Toowoomba, QLD 4350 Australia; 2 School of Accounting, Economics and Finance, University of KwaZulu-Natal, Durban, 4000 South Africa; 3 Murdoch Business School, Murdoch University, Murdoch, WA 6150 Australia; 4 Centre for Health Research and School of Psychology and Wellbeing, University of Southern Queensland, Springfield, QLD 4300 Australia

**Keywords:** Inequity, GINI, Mental health, Need factors

## Abstract

**Background:**

Despite recent substantial mental healthcare reforms to increase the supply of healthcare, mental health inequality in Australia is rising. Understanding of the level of inequity (unmet need gap) in psychiatric service use in Australia’s mixed public–private health care system is lacking.

**Objective:**

To present a novel method to measure inequity in the delivery of psychiatric care.

**Methods:**

Data came from wave 9 (year 2009, *n* = 11,563) and wave 17 (year 2017, *n* = 16,194) of the Household, Income and Labour Dynamics in Australia (HILDA) survey. Multiple logistic regression was employed to estimate the psychiatric care utilisation compared to its need and the Gini index was used to estimate the standardised distribution of utilisation to measure the extent of inequity.

**Results:**

The results show the inequity indices (need-standardised Gini) in psychiatric care utilisation were significant and found to be 0.066 and 0.096 in 2009 and 2017, respectively, for all individuals. In 2009, the inequity indices were found to be 0.051 and 0.078 for males and females, respectively, and 0.045 and 0.068 for rural and urban residents, respectively. In 2017, the indices were calculated to be 0.081 and 0.109 for males and females, respectively, and 0.086 and 0.097 for rural and urban residents, respectively.

**Conclusions:**

This study showed a marked increase in unmet needs in psychiatric care utilisation since 2009. There is a greater need to develop policies to improve equity in psychiatric care utilisation in Australia.

## Introduction

Promoting equity in mental health through need-based provision of psychiatric care is seen as a key objective for many governments. To foster the aim of health equity, the Australian government has introduced the Better Access to Mental Health Care (Better Access) Initiative and the UK government has introduced the Improving Access to Psychological Therapies initiative in recent times [1, 2]. In Australia’s Better Access scheme, a range of mental health services are offered through Medicare (Australia’s national universal health insurance system) and allowed patients in rural and remote areas to access psychological treatment through video conferencing [3]. The scheme has grown in reputation and is initiating interest in the method in countries such as the UK, Canada and New Zealand with similar goals [4]. However, despite the success and increased provision of psychological treatment through the programs, the prevalence of mental disorders had not decreased for many countries and in Australia’s case, recent evidence suggested that mental health inequality had increased [5–7].

Since 1992, under the guidance of National Mental Health Strategy, there has been a substantial reform in mental health service delivery in Australia [8]. In 2018–19, Australia spent $10.6 billion on mental health-related services, a 234% increase in real terms since 1992–93 [9]. Despite these reforms, there has been much debate as to whether they have been effective [10]. Furthermore, questions were raised as to whether the Basaglian de-hospitalised model that Australian policy makers adopted was effective [11, 12]. However, comparative research on such a topic requires a means of measuring inequity that will assesses the health systems’ role in generating inequity. To clarify uncertainties on understanding the Australian mental health system, the current study seeks to present new evidence that could be used to assess the equity impacts of these policy reforms.

While the term inequality and inequity are sometimes confused, they are not interchangeable. Inequity in healthcare refers to unfair and avoidable differences in utilisation of health resources arising from poor governance [13]. On the contrary, inequality in healthcare simply refers to the uneven distribution of utilisation of health resources [13]. Those covered by national health insurance scheme have access to health services more or less equally once they are in the system. However, achieving equity in utilisation of such resources are difficult and requires monitoring of resources for just allocation.

The Australian health system performance with respect to achieving equity in mental health provision is thus not clear. To be exact, questions have been raised as to whether recent policy reforms have improved access to services with respect to need, i.e., whether unmet need is reduced. And if so, to what extent? This paper aims to estimate the level of overall inequity (unmet need gap) in mental health care service utilisation in Australia. We use Wagstaff and colleagues [14] method to estimate inequity.

There are several key areas that this study seeks to contribute to the literature. First, past studies such as that by Meadows and colleagues [15] handled such research questions by estimating the inequality of service utilisation. Although Meadows and colleagues’ work captured some form of disparities through measuring inequalities, it was unable to measure unfairness that stemmed from the need of the patients (i.e., measuring inequality does not capture inequity) [13]. Recent studies clearly revealed that the poor had a 11% higher prevalence of mental disorders than the rich in Australia [16] and thus needs are different among socio-economic strata. Therefore, it is important for research to examine the needs of patients in mental health services utilisation across socioeconomic classes, which this study will do. Second, apart from the Better Access scheme, Australia currently operates more than 30 other mental health programs [17]. Thus, any analysis (for example, Harris et al. [3] and Jorm’s [18] work) that assesses the performance of only the Better Access scheme can be considered partial, compared to the equity perspective of the overall mental health system. Our study will assign Gini index values for the system as a whole and considers the unmet need gap from the overall health system perspective so that it has no such shortcomings.

Furthermore, the recent work of Bartram and Stewarts [19] that compared income inequities of mental health services of Canada and Australia. Bartram and Stewart used Australian National Mental Health and Wellbeing 2007 survey data which was much older data compared with current circumstances. This was especially true, when considered that the Better Access scheme came into operation in November, 2006. Furthermore, Bartram and Stewarts, only compared income inequities and did not compare overall inequity. In this paper, we addressed these issues and details are in the next section.

## Materials and methods

### Data source and sample selection

The analysis of this study is based on the data from the Household, Income and Labour Dynamics in Australia (HILDA) survey [20]. HILDA is a nationally representative household-level longitudinal survey in Australia conducted annually since 2001. HILDA collects data from all individual members of the household who are aged 15 years and older. The questionnaire consists of a main module and a few major and minor sub-modules. The main module of the survey focuses on the socio-demographic characteristics of an individual. These data are obtained through face-to-face interviews and reinterviews of the same selected household members occurs each year. The annual number of survey participants ranges from 12,500 to 17,500 individuals. Over the years, more than 30,000 individuals have participated in face-to-face interviews. On the contrary, the data for major and minor sub-modules are collected from individuals through self-completed questionnaires in different years. Annually, the major and minor sub-modules usually cover a specific theme (for example, wealth, retirement, fertility, health or education) and each theme usually recurs every 3 to 5 years.

The major module covering health appears every 4 years: starting with wave 9 (year 2009), wave 13 (year 2013) and wave 17 (year 2017). For the purpose of this study, we used the waves that contained the earliest (2009) and latest (2017) health modules for our analysis. The principal reason for choosing wave 9 was to use the closest year to the inception of the Better Access scheme and compare it to the most distant year where data are available (wave 17). Only individuals who participated in at least one of wave 9 or 17 were included in the analysis. The final sample contained 19,130 individuals and 27,757 observations (11,563 observations in wave 9 and 16,194 observations for wave 17). Of the 19,130 individuals, 8627 individuals participated in both waves. To account for sample attrition and characteristics of the population, the survey supplied responding person Self-Completion Questionnaire (SCQ) weights have been used for the analysis. In addition, albeit in a very small percentage, non-responses to particular items were imputed using the last observation carry forward method. Detailed information about sampling procedure, wave-on-wave survey response and attrition rates can be found elsewhere [21].

### Measures

#### Outcome variable

The main outcome of interest for this study is psychiatric care utilisation. HILDA collects psychiatric care utilisation data by showing a prompt card on types of health care providers and asking the respondent “During the last 12 months, have you seen any of these types of health care providers about your health?” and if the respondent had a positive response, then the respondent was asked “Which ones?” The response of the respondent who had seen a mental health professional such as a psychiatrist or psychologist was recorded in the HILDA survey as variable “_ hecpmhp”. This binary variable is used as a measure of psychiatric care utilisation in this study.

#### Need and non-need variables

The literature on inequity measurement has mainly developed from Wagstaff and Van Doorslaers’ work [14], based on the horizontal equity principle (that states equal use for equal need). To measure the degree of inequity they standardised the healthcare delivery variable through need and non-need factors. Further empirical work interpreted care delivery as equitable if medical care resources were allocated strictly in accordance with the medical needs of patients and not allocated subjectively to non-need factors such as patient status, income, education or geographic area [22, 23]. Following recent developments in the literature we have classified and selected the need and non-need variables of psychiatric care utilisation for our analysis [24–26]. Medical need in household surveys is not estimable and in practice researchers use demographic and health status/morbidity variables as a proxy of need [23]. This study included the following variables to measure need for psychiatric services: age, gender, general health condition and mental health condition. General health condition of the respondents was assessed using the five-point self-assessed health (SAH) measure included in the SF-36 instrument of the HILDA health module. The mental health condition of a participant was measured using the Kessler Psychological Distress Scale (K10) [27]. The K10 score ranges from 10 to 50 and is used to assess the likelihood of having a mental disorder. For example, a threshold score of 20 or greater indicates the likelihood of developing a mild or above mental disorder depending on how high a person has scored in the K10 scale [28]. Thus, we have defined the binary mental health variable (K10 >  = 20) as a measure for psychiatric care need.

Non-need characteristics such as socio-economic indicators could also have impact in psychiatric care utilisation [19]. Non-need indicators were: income, education, labour force status, socio-economic rank of living area and urbanisation type. The study used equivalised household disposable yearly income in quintiles (poorest, poorer, middle, richer and richest categories) as a measure of income. The ‘modified OECD’ equivalence scale formula was, used to calculate equivalence [29]. The education level was measured as ‘university qualification’ if the participants had an education level above the Australian Qualification Framework (AQF) level 6 (bachelor degree and above), measured as ‘professional qualification’ if AQF level was 3–6 (certificate III–IV, diploma etc.) and measured as ‘12 year or below’ if AQF level was below 3 (year 11–12, etc.) [30]. Labour force status was categorised as employed, unemployed and participants who were not in the labour force (NLF) in the survey period. The socio-economic rank of an area is measured by the 2011 version of Socio-Economic Indexes for Areas (SEIFA) from the Australian Bureau of Statistics (ABS) [31]. This study used SEIFA in quintiles that rank areas of Australia according to relative socio-economic advantage and disadvantage (most disadvantaged areas, disadvantaged areas, median ranked areas, advantaged areas and most advantaged areas). Lastly, the study measured urbanisation type by the 2011 version of the Australian Statistical Geography Standard (ASGS) definition of Section of State (SOS) [32]. If the person lived in a ‘major urban area’ (population greater than 100,000) or ‘other urban area’ (population in between 1000 and 99,999) then the groups were categorised as a person living in urban area in this study. Rest of the population are categorised as a person living in a rural area.

### Statistical analyses

In assessing equity in psychiatric care, our attention focused on assessing the existence of horizontal inequity [33]. The method is fundamentally different from analysing inequality in the utilisation of health care, as equity analysis for health care must account for differences in the need for health care. Thus, according to the horizontal equity principle, variations of utilisation due to need factors are equitable and all other variations are treated as inequitable [23]. A cross-tabulation comparison of psychiatric care need and utilisation distribution by socio-demographic factors would give a general indication of the value judgement of the health care system. However, to assess the extent of inequity, the need standardised distribution of health care utilisation is required to be estimated so that any residual inequality in utilisation can be interpreted as the degree of inequity in utilisation of health care resources.

This study used the indirect standardisation method to measure inequity, which is currently the dominant technique in measuring inequity from household survey data [33]. The steps for estimating need standardised distribution were as follows [22, 23]:

Step 1: the psychiatric care use model that specifies the relationship between psychiatric care use and need/non-need variables is estimated. Since, our outcome of interest is a binary variable (psychiatric care utilisation in the last 12 months—yes/no), we estimate a logistic regression model of the following functional form:

1 $${p}_{i}=\Phi \left({\alpha }_{0}+ {\sum }_{j}{\beta }_{j}{X}_{j}+{\sum }_{k}{\gamma }_{k}{Z}_{k}\right)+ {\varepsilon }_{i},$$

where ${p}_{i}$ is the indicator for psychiatric care use by individual $i$; $\alpha ,\beta \, \&\, \gamma$ are vectors of parameters to be estimated; $X$ is a vector of need variables that we want to standardise (age, gender, general health condition and mental health condition); $Z$ is a vector of non-need variables that we want to control for (income, education, labour force status, socio-economic rank of area and urbanisation types for this study); and ${\varepsilon }_{i}$ is the residual for individual $i$.

Step 2: from the estimated parameters (${\widehat{\alpha }}_{0},{\widehat{\beta }}_{j} \,\& \,{\widehat{\gamma }}_{k}$) of Eq. 1, individual values of need variables (${X}_{j}$) and sample means of non-need variables (${\overline{Z} }_{k}$), we can predict the need-expected utilisation of psychiatric care ${\widehat{p}}_{i}$:

2 $${\widehat{p}}_{i}=\Phi \left({\widehat{\alpha }}_{0}+ {\sum }_{j}{\widehat{\beta }}_{j}{X}_{j}+{\sum }_{k}{\widehat{\gamma }}_{k}{\overline{Z} }_{k}\right)$$

The need-expected utilisation (Eq. 2) predicts the ideal level of psychiatric care an individual would use on average given the same need level, through neutralising the influence of the non-need factors by setting then to their average [22].

Step 3: need-standardised psychiatric utilisation then is derived by subtracting need-expected utilisation from actual psychiatric use and adding the mean of need-expected utilisation. The mean is added so that the mean of the standardised utilisation remains equal to the actual utilisation [23]. Thus, the need-standardised utilisation is as follows:

3 $${\widehat{p}}_{i}^{IS}={p}_{i}-{\widehat{p}}_{i} +\frac{1}{n}\sum_{i=1}^{n}{\widehat{p}}_{i}$$

Step 4: After the estimation of need-standardised utilisation, as in the standard literature, the concentration index is used to measure socioeconomic inequity. We propose that inequity can also be tested by estimating the Gini index of the need standardised utilisation to measure overall inequity (unmet need gap).

Using the above procedure, we can also extend our analysis to compare subgroups (i.e., gender and urbanisation type) for two time periods in Australia. Stata MP Version 15 software and Excel 2016 were used to perform all analyses of this study.

## Results

### Distribution and correlates of psychiatric care use

The distribution of mental illness (individuals who had K10 score 20 or greater) and psychiatric care use by need and non-need factors for 2009 and 2017 are presented in (Table 1). Comparing the rates of mental illness and service utilisation enables an estimate of the shortfall in the healthcare system. Varying degree of service utilisation shortfall exists across all need and non-need factors (i.e., compared with the level of mental illness, service utilisation is low). For example, in 2009, the mental illness rate (as indicated by the proportion of participants reaching the K10 cut-off) for males was 19.13% (95% CI: 17.57–20.78), whereas service utilisation was 4.23% (95% CI: 3.58–5.00). Similarly, for females in the same period, the mental illness rate was 22.88% (95% CI: 21.28–24.56) compared with the service utilisation rate of 6.82% (6.06–7.66). In general, if the mental illness rates were higher for the need factors, the table showed an increase in service utilisation for that factor, although there might still exist varying degree of shortfall.

**Table 1:** Percentage distribution of mental illness and health service utilisation by key socio-demographic characteristics

Characteristics	Observations	2009	Service utilisation	Observations	2017	Service utilisation
		Mental illness			Mental illness	
	(*N*)	(%) (95% CI)	(%) (95% CI)	(*N*)	(%) (95% CI)	(%) (95% CI)
*Need factors*						
Age						
15–24 years	2137	25.54 (23.16–28.07)	5.54 (4.40–6.94)	2588	34.87 (32.40–37.43)	12.44 (10.80–14.28)
25–39 years	2735	22.05 (19.60–24.70)	6.59 (5.55–7.82)	4217	28.43 (25.92–31.09)	11.26 (9.79–12.92)
40–64 years	4791	20.15 (18.35–22.08)	6.19 (5.38–7.10)	6289	23.24 (21.54–25.03)	8.51 (7.59–9.54)
65 + years	1900	16.38 (13.79–19.35)	2.08 (1.42–3.03)	3100	15.59 (13.11–18.45)	2.72 (2.15–3.42)
Gender						
Male	5400	19.13 (17.57–20.78)	4.23 (3.58–5.00)	7601	22.85 (21.37–24.41)	7.16 (6.39–8.02)
Female	6163	22.88 (21.28–24.56)	6.82 (6.06–7.66)	8593	27.36 (25.82–28.97)	10.46 (9.50–11.49)
Self-Assessed Health						
Excellent	1505	6.61 (4.93–8.82)	2.95 (1.97–4.39)	1825	9.7 (7.73–12.11)	4.66 (3.58–6.03)
Very good	4285	12.64 (10.92–14.59)	3.90 (3.19–4.76)	5633	15.74 (14.39–17.21)	6.49 (5.56–7.56)
Good	3980	23.32 (21.51–25.23)	5.41 (4.58–6.38)	5817	28.01 (25.99–30.13)	9.29 (8.19–10.52)
Fair	1439	41.15 (37.52–44.87)	11.09 (9.19–13.33)	2375	43.34 (40.41–46.32)	13.30 (11.68–15.11)
Poor	354	63.43 (54.65–71.40)	12.39 (8.83–17.13)	544	68.03 (63.12–72.58)	23.87 (19.77–28.51)
*Non-need factors*						
Household yearly disposable income quintile						
Poorest	2314	31.54 (28.22–35.05)	6.90 (5.62–8.44)	3239	32.23 (30.10–34.44)	10.16 (8.82–11.69)
Poorer	2313	22.85 (20.41–25.49)	6.25 (5.05–7.71)	3239	29.13 (25.21–33.39)	9.05 (7.67–10.64)
Middle	2311	20.38 (18.12–22.84)	5.38 (4.35–6.65)	3239	26.97 (24.01–30.09)	8.53 (7.38–9.85)
Richer	2313	17.74 (15.41–20.35)	4.60 (3.62–5.83)	3239	20.66 (18.66–22.81)	8.13 (6.71–9.81)
Richest	2312	13.59 (11.64–15.82)	4.69 (3.75–5.85)	3239	17.56 (15.72–19.62)	8.48 (7.25–9.89)
Education						
Year 12 or below	5646	24.76 (22.89–26.73)	5.38 (4.66–6.21)	6501	30.18 (28.32–32.1)	9.81 (8.82–10.90)
Professional Qualification	3307	19.06 (17.08–21.21)	5.46 (4.57–6.50)	5357	24.89 (23.08–26.79)	8.43 (7.58–9.39)
University Qualification	2570	14.70 (12.97–16.62)	6.03 (4.91–7.39)	4336	17.99 (16.02–20.14)	7.84 (6.68–9.17)
Labour force status						
Employed	7435	17.15 (15.87–18.51)	4.68 (4.08–5.37)	10,254	22.35 (21.21–23.54)	8.04 (7.24–8.92)
Unemployed	395	38.41 (32.12–45.11)	13.43 (9.29–19.01)	628	47.42 (38.09–56.94)	16.13 (12.17–21.08)
Not in Labour Force (NLF)	3733	26.60 (24.17–29.18)	6.33 (5.42–7.39)	5312	28.12 (26.11–30.23)	9.58 (8.56–10.71)
SEIFA						
Most disadvantaged	2315	30.07 (26.62–33.77)	6.08 (4.82–7.64)	3239	31.26 (28.48–34.18)	8.80 (7.22–10.70)
Disadvantaged	2312	21.89 (19.45–24.53)	4.66 (3.64–5.95)	3252	26.26 (24.37–28.25)	8.70 (7.51–10.06)
Median	2317	20.41 (18.02–23.02)	5.69 (4.67–6.91)	3242	25.71 (23.10–28.50)	9.45 (8.20–10.86)
Advantaged	2308	16.89 (14.85–19.15)	5.55 (4.51–6.81)	3223	21.88 (19.74–24.18)	9.50 (8.11–11.09)
Most advantaged	2311	16.08 (14.26–18.10)	5.67 (4.55–7.04)	3238	21.53 (17.94–25.61)	7.85 (6.56–9.36)
Urbanisation types						
Urban	10,056	21.29 (19.90–22.74)	5.84 (5.26–6.48)	14,095	25.48 (24.09–26.92)	9.06 (8.35–9.81)
Rural	1507	18.73 (16.30–21.43)	2.88 (1.99–4.16)	2099	22.54 (19.72–25.63)	7.10 (5.81–8.64)

The same is not true for some non-need factors such as income, education and socioeconomic rank by area. Whereas, the mental illness rates between groups were significantly different for these factors, the service utilisation rates were not significantly different. For example, in 2017, the poorest income group had 32.23% (95% CI: 30.10–34.44) mental illness rate and the richest income group had a rate of 17.56% (95% CI: 15.72–19.62). Clearly, the data show that individuals belonging to higher income groups had significantly lower mental illness rates. However, the poorest income group had 10.16% (95% CI: 8.82–11.69) utilisation rate and richest income group had 8.48% (95% CI: 7.25–9.89) despite the large difference in mental illness rates.

In summary, (Table 1) shows that higher mental illness is matched with higher utilisation rates across all need factors and some non-need factors. In addition, both illness and utilisation rates were higher in 2017 than in 2009. However, a service utilisation shortfall exists in all factors in both years. Table 1 indicates the existence of inequity in psychiatric care utilisation, but fails to confirm and measure the extent of inequity in psychiatric care. To understand the level of inequity, we need to investigate the estimates of the logistic regression model.

(Table 2) reports the need-expected correlates of psychiatric care utilisation for 2009 and 2017. Unsurprisingly, people who were more likely to be mentally ill (K10 score 20 or greater), had the highest odds of psychiatric care utilisation among all factors (adjusted odds ratio [AOR]: 3.883 and 4.321 for years 2009 and 2017, respectively). Similarly, individuals who reported their health (Self-Assessed Health) as ‘fair’ or ‘poor’ had higher odds to use psychiatric services compared to individuals who reported their health as ‘excellent’ (AOR: 2.984 and 2.707 in 2009 and 2.481 and 3.933 in 2017, respectively, for ‘fair’ and ‘poor’). In addition, the results showed that women had higher odds (AOR: 1.55 and 1.39) than men in using psychiatric care in 2009 and 2017, respectively. The results also showed that the older age group (65 + years) had significantly lower odds (AOR: 0.247 and 0.158, respectively, in 2009 and 2017) of psychiatric care utilisation than the reference age group (15–24 years). Thus, the need type variables showed expected patterns in the logistic regression model results.

**Table 2:** Correlates of healthcare service utilisation (Logistic regression models)

Characteristics	Unadjusted OR	2009	Adjusted OR	95% CI	Unadjusted OR	2017	Adjusted OR	95% CI
		95% CI				95% CI		
Age								
15–24 years (ref.)								
25–39 years	1.204	(0.894–1.622)	1.2	(0.861–1.673)	0.893	(0.723–1.104)	1.006	(0.794–1.274)
40–64 years	1.125	(0.857–1.477)	1.028	(0.740–1.430)	0.655***	(0.542–0.791)	0.668***	(0.539–0.826)
65 + years	0.362***	(0.234–0.561)	0.247***	(0.143–0.427)	0.197***	(0.148–0.261)	0.158***	(0.113–0.220)
Gender								
Male (ref.)								
Female	1.655***	(1.343–2.039)	1.546***	(1.236–1.935)	1.514***	(1.298–1.766)	1.387***	(1.175–1.639)
Self-Assessed Health								
Excellent (ref.)								
Very good	1.335	(0.85–2.097)	1.247	(0.776–2.004)	1.421*	(1.042–1.939)	1.347	(0.983–1.847)
Good	1.880**	(1.200–2.945)	1.498	(0.934–2.401)	2.098***	(1.582–2.782)	1.779***	(1.336–2.370)
Fair	4.105***	(2.614–6.446)	2.984***	(1.834–4.854)	3.142***	(2.318–4.260)	2.481***	(1.797– 3.427)
Poor	4.653***	(2.668–8.114)	2.707**	(1.44–5.090)	6.42***	(4.484–9.193)	3.933***	(2.666– 5.800)
Mental Illness (K10 > = 20)								
No (ref.)								
Yes	5.330***	(4.273–6.649)	3.883***	(3.076–4.903)	6.128***	(5.136–7.311)	4.321***	(3.596– 5.191)
Household yearly disposable income quintile								
Poorest (ref.)								
Poorer	0.9	(0.655–1.236)	1.044	(0.715–1.524)	0.879	(0.697–1.108)	0.859	(0.672– 1.098)
Middle	0.768	(0.556–1.059)	0.868	(0.584–1.289)	0.825	(0.666–1.022)	0.868	(0.668–1.129)
Richer	0.651**	(0.470–0.902)	0.756	(0.515–1.110)	0.782	(0.604–1.013)	0.966	(0.721–1.295)
Richest	0.664**	(0.487–0.906)	0.781	(0.527–1.158)	0.819	(0.646–1.037)	1.135	(0.863–1.493)
Education								
Year 12 or below (ref.)								
Professional Qualification	1.015	(0.803–1.283)	1.259	(0.969–1.637)	0.847*	(0.732–0.980)	0.946	(0.804–1.112)
University Qualification	1.129	(0.864–1.476)	1.441*	(1.073–1.935)	0.781*	(0.638–0.956)	0.983	( 0.754–1.283)
Labour Force Status								
Employed (ref.)								
Unemployed	3.155***	(2.030–4.903)	2.461***	(1.492–4.059)	2.199***	(1.558–3.105)	1.452	(0.969– 2.175)
Not in Labour Force (NLF)	1.376**	(1.106–1.712)	1.379*	(1.051–1.811)	1.212*	(1.027–1.430)	1.49***	(1.226–1.812)
SEIFA								
Most Disadvantaged (ref.)								
Disadvantaged	0.755	(0.529–1.078)	0.945	(0.641–1.395)	0.987	(0.749–1.301)	1.197	(0.861–1.663)
Median	0.931	(0.674–1.286)	1.397	(0.970–0.012)	1.081	(0.834–1.4)	1.338	(0.976–1.834)
Advantaged	0.907	(0.655–1.255)	1.534*	(1.060–2.219)	1.087	(0.829–1.425)	1.532**	(1.107–2.121)
Most advantaged	0.928	(0.664–1.297)	1.565*	(1.059–2.314)	0.882	(0.669–1.162)	1.201	(0.836–1.725)
Urbanisation types								
Urban (ref.)								
Rural	0.479***	(0.322–0.712)	0.491***	(0.324–0.744)	0.767***	(0.612–0.962)	0.772*	(0.603–0.988)
Constant			0.013***	(0.007–0.025)			0.028***	(0.018–0.044)

***p < 0.001, **p < 0.01, and *p < 0.05

This study did not find any evidence that non-need factors such as income or socio-economic area ranks (SEIFA) had any significant association with psychiatric care utilisation. This is understandable since patients are supported through Medicare, the national health insurance scheme. However, the study found that individuals with university levels of education qualification used significantly more psychiatric care (AOR: 1.441) compared to individuals who had lower levels of education in 2009. Similarly, individuals who were unemployed or not in the labour force (NLF) used significantly higher levels of psychiatric care (AOR: 2.461 and 1.379, respectively, for unemployed and NLF in 2009 and 1.49 for NLF in 2017) than employed individuals. Although, the education and unemployed groups were not significant in 2017. The results also show that non-need factors such as urbanisation types were significant in both years. Individuals who reside in a rural area had lower odds of psychiatric care utilisation than individuals who lived in urban areas (AOR: 0.013 and 0.028 in 2009 and 2017 respectively for rural areas). While regression estimates showed the relative importance of each factor, it did not show the extent of inequity in psychiatric care utilisation in Australia. For that, we have to use the regression estimates to generate a need-standardised distribution and use the inequality indices of the distribution to measure inequity.

### Inequity in psychiatric care utilisation

The levels of inequity and inequality of psychiatric care service utilisation in Australia are presented in (Table 3). The socioeconomic inequality and inequity indices (measured by concentration index) are bounded between -1 and 1. A negative value indicates pro-poor inequity/inequality and a positive value indicates pro-rich inequity/inequality of utilisation of healthcare. Conversely, overall inequality and inequity indices (measured by the Gini index) are bounded between 0 and 1. In one extreme, a zero value indicates equal distribution to all and on the other extreme a value of one indicates highest levels of unequal distribution of healthcare utilisation (all psychiatric care is utilised by only one person). (Table 3) shows that there exists significant level of pro-poor socioeconomic inequality of − 0.087 in 2009. However, this inequality was lower but not significant in 2017. Furthermore, socioeconomic inequity (when need was standardised) in both 2009 and 2017 was not significant, implying there was no socioeconomic inequity in the study period.

**Table 3:** Inequity and inequality of psychiatric healthcare service utilisation

	2009	2017
Socioeconomic inequality	− 0.087**	− 0.034
Socioeconomic inequity	− 0.002	0.001
Overall inequality	0.945***	0.912***
Overall inequity	0.066***	0.096***

***p < 0.001, **p < 0.01, and *p < 0.05

To understand the inequity level further, we also studied overall inequality and inequity. Overall inequality and inequity were significant in both years. The overall inequality for psychiatric care utilisation was 0.945 and 0.912 in 2009 and 2017, respectively. By 2017, the level of inequality was reduced, i.e., more individuals had used psychiatric care in 2017. However, the simple inequality measurement does not account for the need of health care. Individuals with greater need will obviously use higher levels of healthcare if available. Inequity measurement takes into account an individuals’ need/non-need factors and indicates the level of unfairness in healthcare utilisation. (Table 3) shows that overall inequity in psychiatric care utilisation were 0.066 and 0.096 in 2009 and 2017, respectively (statistically significant in both years). Contrary to overall inequality, the inequity level in psychiatric care utilisation in Australia had risen by 2017.

**Fig. 1:**
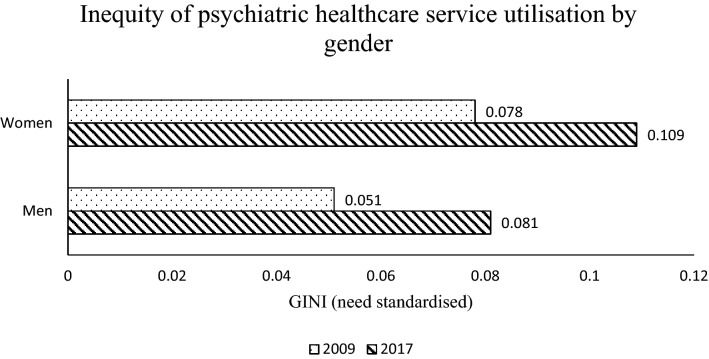
Inequity of psychiatric healthcare service utilisation by gender in 2009 and 2017

Figure 1 shows the gender specific inequity level of psychiatric services in 2009 and 2017. The inequity levels of psychiatric care utilisation for women are 0.078 and 0.109 in 2009 and 2017, respectively. Conversely, the levels for men are 0.051 and 0.081 in 2009 and 2017, respectively. Women experience a higher level of inequity in psychiatric care utilisation than men in Australia. The inequity level also increased for both genders by 2017.

**Fig. 2:**
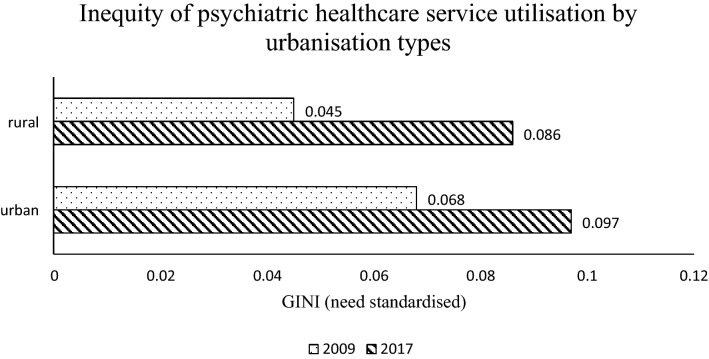
Inequity of psychiatric healthcare service utilisation by urbanisation types in 2009 and 2017

The inequity level also varies by area. Figure 2 shows the inequity level by urbanisation. Individuals who lived in rural areas encountered lower level of inequity in psychiatric care utilisation than individuals who lived in urban areas (0.045 and 0.086, respectively, in 2009 and 2017 for rural residents and 0.068 and 0.097, respectively, in 2009 and 2017 for urban residents). The inequity level had increased for both types of residents in 2017 and the inequity gap between these two groups reduced.

## Discussion

Gaps continue to exist in the literature when estimating the degree of unmet needs in the mental healthcare system. Although most studies report correlates of unmet need of mental healthcare utilisation, it is difficult to draw precise comparison across health systems because such studies lack a standardised measure of unmet need [34–36]. However, in the health inequity literature, the CI approach has been used to measure the extent of disparity in mental health need and its utilisation in different socioeconomic strata through the use of an inequity index [37]. Similar to the CI approach, we proposed that the Gini index could be used to measure the degree of unmet mental health needs. The study findings demonstrated marked inequity in mental healthcare service provision even after the introduction of the Better Access scheme in Australia.

Contrary to previous studies, our study found that there was no significant socioeconomic inequity in mental health care use, i.e., we did not find any significant disparity in mental healthcare use among different socioeconomic groups after we adjusted for needs and non-need factors [15, 19]. First, this contradiction likely arose, because Meadows et al. [15] did not adjust for need-factors in their work. Second, Bartram and Stewart [19] used old data that did not correspond to current healthcare provision in Australia. Furthermore, Bartram and Stewart investigated mental health service providers (psychiatrist, psychologist, etc.) separately and as patients can avail services with different providers, the result could very well be different if their analysis was conducted in an aggregate system level.

However, our results are consistent with the findings of Harris et al. [3] and Jorm [18] that confirm that there exists marked unmet need in Australia’s mental health delivery system. However, unlike Harris et al. and Jorm’s works which evaluated the Better Access scheme specifically, our work evaluated the mental health system as a whole and found that there was a 45% increase in index score (unmet need gap) in recent times despite the introduction of the Better Access scheme.

Previous research suggested that women have higher needs and are more likely to use mental health care than men [38]. This situation is also confirmed in our study. The findings showed that at a population level, females’ unmet need is higher than males even after adjusting for need and non-need factors and holds in both periods. Thus, policy makers might need to design and implement strategies focusing higher levels of service delivery to female populations so that such unmet need is reduced. Our study findings also suggest that unmet need is higher in urban areas than rural areas in Australia. The Australian government should be commended for mental health service delivery in rural areas. However, the rate of increase in unmet need in rural areas was higher when compared to urban areas. The Australian government should formulate policy targets that cost effectively increase need based psychiatric care access in the rural areas.

It is important to consider certain limitations of our study findings. First, instead of the perceived needs of an individual, this study used K10 self-report surveys and self-assessed health to measure the need for psychiatric utilisation. Thus, it is possible that it excludes those who need care but do not fulfil the clinical cut off criteria, for example, those with sub-clinical symptoms or those seeking early intervention or assistance with wellbeing. However, at the population level, the clinical cut-offs associated with the K10 provide a good indication of the proportion of people who would indicate a need for some type of psychiatric assistance. Second, if data were available before 2007, then it would be possible to investigate the health reform effect of programs like Better Access scheme more accurately. Further, data unavailability limits our analysis to only 2 years (2009 and 2017) which fails to capture the within year effects that a trend analysis would have allowed. Finally, it might also be beneficial to examine service utilisation in a more detailed manner, for example, how many times participants had utilised psychiatric services rather than simply whether these services had been accessed during the time period. This could explain finer details of socioeconomic inequity. However, we could not perform such analysis, because of data unavailability. Given these limitations, this study calls for prospective research and future surveys that capture changes in the level of unmet need over time for countries that have similar equity objectives.

## Conclusions

Service equity across socio-demographic characteristics, regions and communities is one of the primary goals of Australia’s National Mental Health Strategic Plan. Despite recent mental healthcare reforms, our results showed that equity has not been fully achieved in psychiatric care delivery in Australia. Although our results did not find any significant socioeconomic inequity in mental healthcare use, they suggest that there is an unmet need gap which is increasing across all communities in Australia. Furthermore, there is a need for policies to address the unmet needs of psychiatric care for women. Although Australia’s health care system performs well compared to the rest of the world, there is a need to focus on improving equity and efficiency performance of existing policies and help develop targeted strategies that improve the equity of psychiatric care for all Australians.

## Figures and Tables

**Fig. 3:**
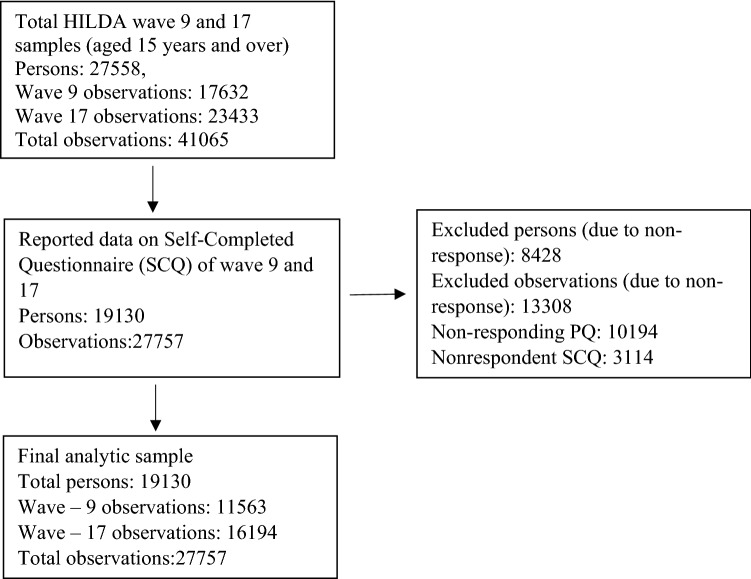
Participants flow into the sample

## Data Availability

The data are available from the National Centre for Longitudinal Data (NCLD) of DSS for researchers of approved organisations who meet the criteria for access to confidential data.

## Appendix

See Fig. 3.

## Code availability

The programming code for analyses in the study can be made available upon request as determined by the authors.
